# The protective or damaging effect of Tumor necrosis factor-α in acute liver injury is concentration-dependent

**DOI:** 10.1186/s13578-016-0074-x

**Published:** 2016-02-03

**Authors:** Yulong Dong, Yuzhou Liu, Xingrui Kou, Yingying Jing, Kai Sun, Dandan Sheng, Guofeng Yu, Dandan Yu, Qiudong Zhao, Xue Zhao, Rong Li, Mengchao Wu, Lixin Wei

**Affiliations:** Tumor Immunology and Gene Therapy Center, Eastern Hepatobiliary Surgery Hospital, The Second Military Medical University, Shanghai, China; Central Laboratory of Medical Research, Renji Hospital, Shanghai Jiaotong University School of Medicine, Shanghai, China

**Keywords:** TNF-α, Enbrel, CCl_4_, Liver injury

## Abstract

**Background:**

Inflammatory cytokine is important in modulating injured diseases. Tumor necrosis factor-α (TNF-α), one of potent inflammatory cytokines, plays a dominant role in host defense reaction. However, the concrete effect of TNF-α on acute liver injury is totally unclear. Here we reported the concrete effect and possible mechanisms of TNF-α on acute liver injury induced by carbon tetrachloride (CCl_4_).

**Methods:**

SD male rats were equally divided into nine groups. CCl_4_ (1 ml/kg) was subcutaneously injected into the rats. Enbrel, a TNF-α inhibitor, were intraperitoneally injected at dose of 0, 0.25, 0.5, 1, 2, 4 or 8 mg/kg 15 min before the CCl_4_ injection. 24 h later, rats were sacrificed. Serum ALT and AST were measured with an autoanalyzer. Serum TNF-α were measured by ELISA. HE staining was used to observe the liver tissue morphology. Hepatocellular apoptosis were tested by immunochemistry and Tunnel kit. Inflammatory factors, involve IL-4, IL-6, IL-8, IL-β and IFN-γ were detected by RT-PCR. The NF-κB signal pathway and anti-apoptotic genes include Bcl-XL, FHC, XIAP and Bcl-2 were measured by western-blotting and RT-PCR.

**Results:**

The change of liver function presented an obvious “V” shape in the whole process of persistently increased Enbrel. As Enbrel was increased gradually from 0 to 1 mg/kg, serum TNF-α were blocked, ALT and AST were gradually decreased as TNF-α as well as the numbers of hepatocellular apoptosis, and were declined to the minimum at 1 mg/kg Enbrel. As Enbrel was increased gradually from 1 to 8 mg/kg, ALT, AST and hepatocellular apoptosis were increased instead, and reached to the maximum at 8 mg/kg Enbrel. HE showed that the seriousness of hepatocellular steatosis was the most at 8 mg/kg Enbrel, and second at 0 mg/kg, the weakest at 1 mg/kg in the acute liver injury. Western-blotting and RT-PCR showed NF-κB, p-IκBα and antiapoptotic genes include Bcl-XL, FHC, XIAP, Bcl-2 were decreased as TNF-α was blocked by increased Enbrel.

**Conclusion:**

Our results suggested that TNF-α had a dual role in acute liver injury. It was regulated might via the corporate effect of NF-κB signal pawahway and anti-apoptosis. Meanwhile, our findings provide a reference for clinical treatment of acute liver injury.

**Electronic supplementary material:**

The online version of this article (doi:10.1186/s13578-016-0074-x) contains supplementary material, which is available to authorized users.

## Background

Liver is a specialized organ in terms of its metabolic, synthetic and detoxifying function. It is a vulnerable organ as various noxious agents can be assimilated by intestine and transported into liver, inducing inflammation, necrosis, fibrosis, cirrhosis [1] and eventually hepatocellular carcinoma (HCC). Hepatic ischemia–reperfusion injury [2], endotoxemia [3], obstructive cholestasis [4], alcoholic hepatitis [5], halothane hepatitis [6], and hemorrhagic shock [7] can cause liver injury. Over 80 % of HCCs occur in patients with hepatic cirrhosis or fibrosis and thus develop in a setting of hepatocellular injury, regeneration, infiltration of inflammatory cells and an abundance of activated myofibroblasts [8]. Despite different reasons, initiation of hepatocellular injury is the key to the common pathogenesis described previously, which in the long run promote the development of hepatic fibrosis and HCC [9]. Unfortunately, at present, the exact mechanisms of liver injury are still under study and yet, the treatment for liver injury is still a big problem to human. Carbon tetrachloride (CCl_4_), a kind of hepatotoxin, can effectively duplicate liver injury model caused by its production of cytochrome P450 and the binding reaction with intermediate metabolites [10, 11]. It provides beneficial foundation for further studying liver injury.

However, in the pathological process of liver injury, numerous inflammatory cytokines are significantly upregulated, such as tumor necrosis factor-α (TNF-α), which mediate inflammation and repair in physiological conditions [12]. Tumor necrosis factor-α is a multifunctional pro-inflammatory cytokine that regulates multifarious processes including inflammation, cellular apoptosis, coagulation, metabolism, insulin sensitivity, tumor growth and invasion, and vascular functions [13–17]. Normally, TNF-α is required for hepatocyte proliferation during liver regeneration induce the transcription factor nuclear factor-κB, which has protective effects [18]. After injury, even when no infectious agents are present, TNF-α quickly migrate into the injured tissue after vasodilatation and suppress further cell death, activate stem cells, and promote epithelial proliferation [19]. However, on the other hand, some literatures have reported TNF-α paly a malignant role in the injured liver. It triggers a series of intracellular events that result in activation of apoptosis and accelerating hepatic cells death during liver injury [20]. And elevated serum levels of TNF-α in liver injured patients correlate with a detrimental prognosis [20, 21].

Despite current knowledge of TNF-α, litter is reported the concrete role of TNF-α in acute liver injury. Moreover, the influence of different concentration of TNF-α mediated by Enbrel (Etanercept), which is a genetically engineered fusion protein that can bind and inactivate TNF-α [22], on CCl_4_-induced acute liver injury remains largely unknown. Our present study is designed to determine the concrete effect of TNF-α on acute liver injured model induced by CCl_4_ and possible mechanisms associated with it.

## Results

### TNF-α performs protective and harmful effects in CCl_4_-induced acute liver injury

**Fig. 1:**
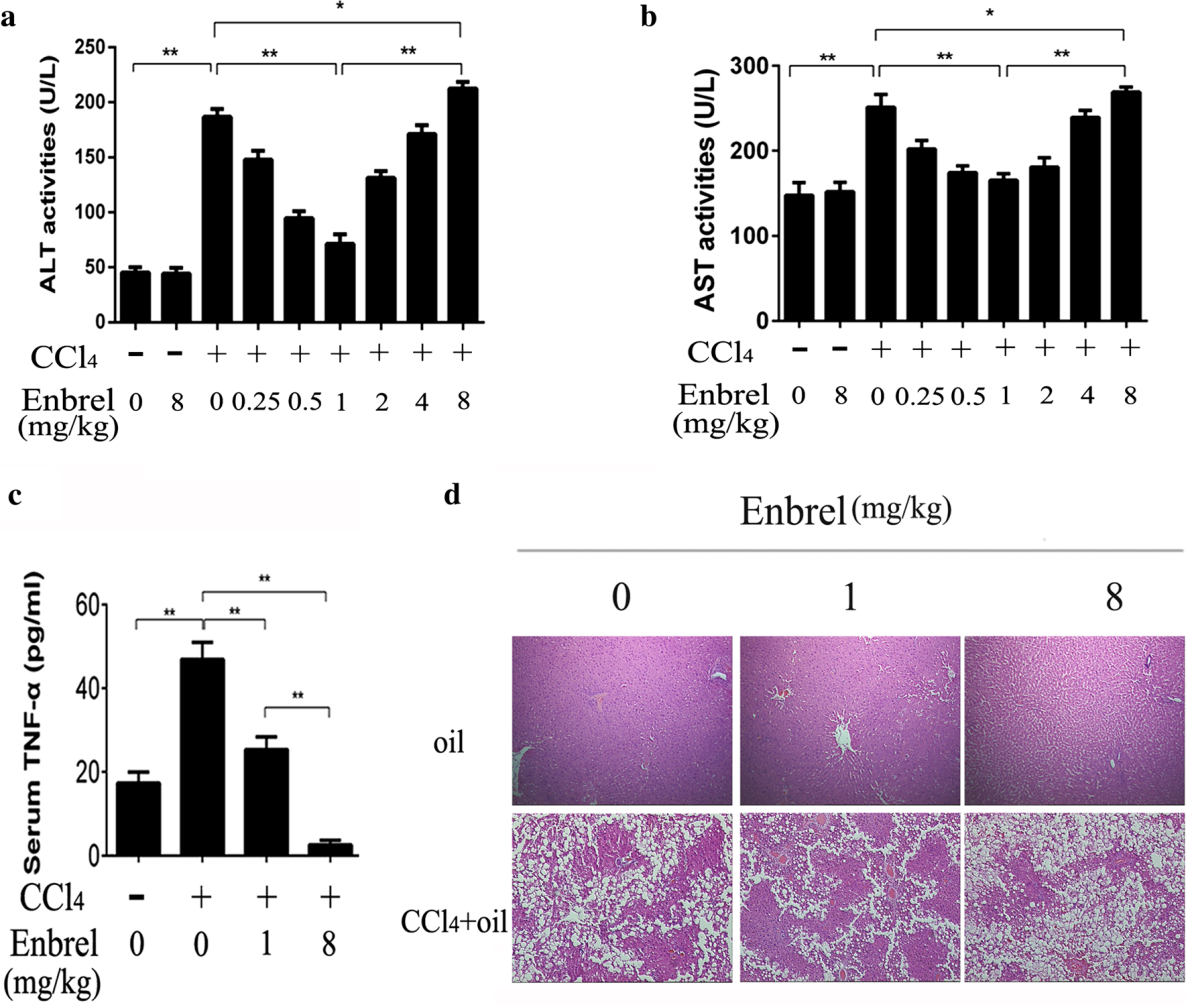
Different levels of internal TNF-α influence on the CCl_4_-induced acute liver injured model. **a** Serum ALT and **b** AST levels were examined by Roche Diagnostic kits in Hithachi Modular P Autoanalyser. The levels of ALT and AST were significantly elevated 24 h later after subcutaneous injection of 1 ml/kg CCl_4_ into Sprague–Dawley rats. (ALT: from 36.75 ± 8.73 to 193.27 ± 14.26 U/L; AST: from 150.86 ± 12.34 to 253.79 ± 12.90 U/L. p < 0.01). In contrast to the CCl_4_ group, the ALT and AST levels were all down-regulated as Enbrel were administrated at doses of 0.25, 0.5 and 1 mg/kg, and elevated again at dose of 2, 4 and 8 mg/kg 15 min before injection of the CCl_4_. Data were expressed as mean ± SD of seven rats for each group. *p < 0.05, **p < 0.01. **c** ELISA assay of serum TNF-α was notably elevated after CCl_4_ administered, and gradually decreased as the doses of Enbrel were added. **d** Hematoxylin-eosin–stained liver sections from control and CCl_4_-induced rats were pretreated with different concentrations of Enbrel (scale bar, 100 μm). In the CCl_4_-induced acute liver injury, the hepatocellular steatosis were serious especially at 0 and 8 mg/kg Enbrel treatment, and the structures of hepatic lobule were disorder, companied with hepatocyte degeneration and inflammatory cells infiltrating. In the oil-treated rats no matter much Enbrel was pretreated, there were litter hepatocellular steatosis, and the structures of hepatic lobule were clear, with few degenerated liver cells and infiltrated inflammatory cells

To investigate the effects of TNF-α on acute liver injury, different doses of Enbrel were pretreated 15 min before CCl_4_ administered. Serum alanine aminotransferase (ALT) and aspartate transaminase (AST) were significantly elevated after subcutaneous injection of CCl_4_. The ALT and AST levels were all decreased pre-treated with Enbrel of 0.25, 0.5 and 1 mg/kg when the serum TNF-α was slightly blocked (Fig. 1c) in CCl_4_-induced liver injured rats (Fig. 1a, b). Our data suggested ALT had 62 % reduction and AST had 33 % reduction as Enbrel was added from 0 to1 mg/kg. The serum ALT and AST were dropped to the minimum at 1 mg/kg Enbrel. It implied that TNF-α had some damaging effect in acute liver injury, and appropriately down-regulated TNF-α level would alleviate the injury. However, as TNF-α was sequentially blocked at high dose of Enbrel (4 and 8 mg/kg), the ALT and AST levels were increased instead and reached the maximum at 8 mg/kg Enbrel when litter serum TNF-α was detected (Fig. 1c). In the futher research of the function, we found the similar results in TNF-α deleted rats. As TNF-α was deficient, the liver injury was significantly aggravated after administration of CCl_4_ (Additional file 1). It might hint that TNF-α had some protective effect in the acute liver injury. And its protective role was gradually decreased as TNF-α level was declined. However, interestingly, the serum ALT and AST were less at 0 mg/kg Enbrel used than 8 mg/kg Enbrel. It indicated that the extent of hepatocellular damage were slighter at high level of TNF-α (0 mg/kg Enbrel) than very low level of TNF-α (8 mg/kg Enbrel). Thus, high concentration of TNF-α also had some protective role for the hepatocytes in the acute liver injury. Furthermore, Hematoxylin and Eosin (HE) staining results also showed that the hepatocellular steatosis was serious at 0 or 8 mg/kg Enbrel, especially more serious at 8 mg/kg Enbrel used in CCl_4_-induced acute injury rats (Fig. 1d). The hepatocellular damage was alleviated at 1 mg/kg Enbrel used. These results suggested that TNF-α might play a dual role in the CCl_4_-induced liver injury.

### The apoptosis of liver cells in acute liver injured rats

**Fig. 2:**
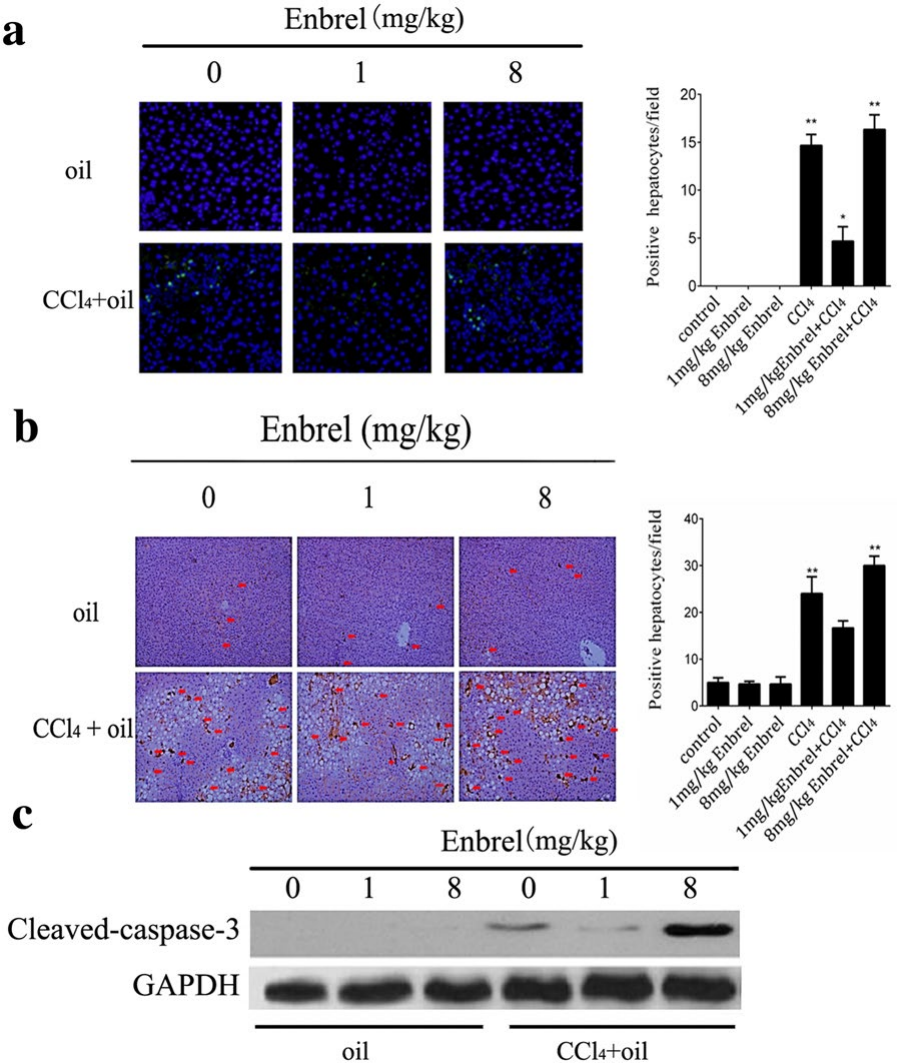
TNF-α influences hepatocellular apoptosis in CCl_4_-induced acute liver injury. **a** TUNEL assay was performed using and **b** immunohistochemistry analyzed of hepatocellular apoptosis in CCl_4_-induced rats with anti-cleaved caspase-3 (magnification ×400). There was litter apoptosis as different doses of Enbrel were injected into the normal rats. Appropriately reduced TNF-α level would decrease hepatocellular apoptosis, and excessively reduced TNF-α level would aggravate apoptosis in CCl_4_-induced-acute liver injury. The *arrows* represented apoptotic cells. **c** Western-blot analyzed the expression of hepatocellular apoptosis by detecting cleaved-caspase-3 in CCl_4_-induced rats. Number of apoptotic hepatocytes was reduced at appropriately low level of TNF-α, while increased at high and excessive low level of TNF-α in acute liver injured rats. (*p < 0.05, **p < 0.01)

Injury is typically associated with changes of apoptosis, and TNF-α is also known to link with apoptosis [13], which promoted us to study the relationship between TNF-α and apoptosis in CCl_4_-induced rats. To determine the relationship between hepatocellular apoptosis and TNF-α level, TUNEL assay and the expressions of cleaved-caspase-3, an apoptotic protein, were detected by immunohistochemistry (Fig. 2a, b). We found there were almost no hepatocellular apoptosis during the process of increasing doses of Enbrel in the rats without administration of CCl_4_. It indicated that changed TNF-α level would not harmful for hepatocytes in the healthy rats. However, after the treatment of CCl_4_, high level of TNF-α was obviously increased as well as hepatocellular apoptosis at 0 mg/kg Enbrel. Meanwhile, as Enbrel was increased to 1 mg/kg, the apoptosis were significantly decreased. And they were elevated instead when Enbrel was added from 1 to 8 mg/kg. Next, we detected the cleaved-caspase3 expression by Western-Blotting. As we predicted that cleaved-caspase-3 expression was high at 0 and 8 mg/kg Enbrel, and was very low at 1 mg/kg in CCl_4_-induced rats. Meantime, it was clearly that expression of cleaved-caspase3 at 8 mg/kg was stronger than 0 mg/kg (Fig. 2c). These results further suggested that TNF-α had a dual role in the CCl_4_-induced liver injury. Hepatocytes were effectively protected as concentration of TNF-α was appropriately reduced, and aggravated as TNF-α excessively reduced to a low level.

### Internal concentration of TNF-α had influence on expression of inflammatory factors in CCl_4_-induced acute liver injury

**Fig. 3:**
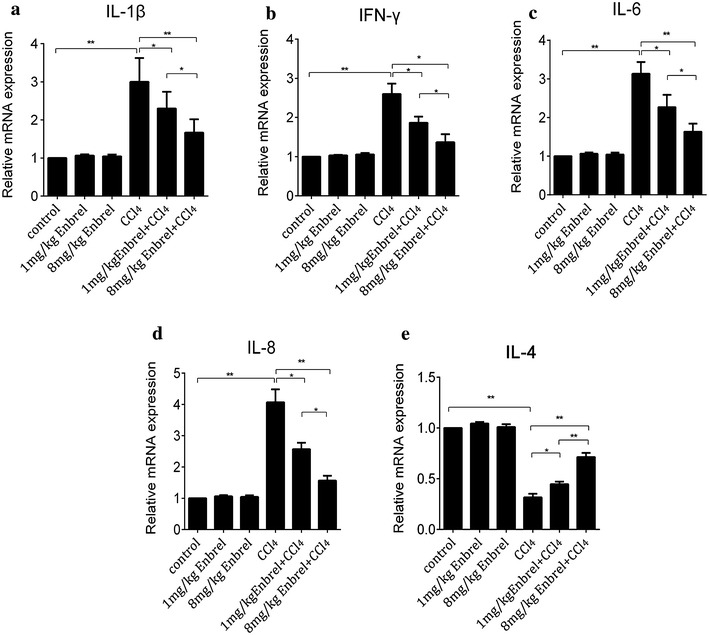
Expressions of inflammatory factors were related with TNF-α. RT-PCR mRNA analysed the expression of **a** IL-1β, **b** IFN-γ, **c** IL-6, **d** IL-8, and **e** IL-4 in CCl_4_-induced rats. We set mRNA expression of these inflammatory factors as one in each control groups. (*p < 0.05, **p < 0.01)

As TNF-α is a potent pro-inflammatory cytokine, and injury is often accompanied with inflammation. Then we next studied the influence of TNF-α on inflammation in CCl_4_-induced rats. Several inflammatory factors, involve IL-4, IL-6, IL-8, IL-β and IFN-γ were detected at different concentrations of TNF-α in common and CCl_4_-induced rats. RT-PCR showed that mRNA expressions of inflammatory factors were dose-dependent on internal TNF-α level in acute liver injury, but litter influence on common rats without injection of CCl_4_, no matter how much the level of TNF-α were changed. It implied that decreased TNF-α would hardly affect expression of these inflammatory factors in common rats. However, in CCl_4_-induced rats, the expression of IL-6, IL-8, IL-β and IFN-γ were decreased as the concentration of TNF-α was blocked by increased dose of Enbrel, (Fig. 3a–d) while IL-4 had a completely opposite outcome (Fig. 3e). This because IL-4 is secreted by Th2 cells, and block Th1 cells producing IFN-γ,IL-6 and TNF-α [23] et al. It might suggest that TNF-α could mediate inflammatory response and promote expression of inflammatory factors in acute liver injury. However, the expression of IL-6, IL-8, IL-β and IFN-γ were still inhibited at excessively low level of TNF-α. It seemed that the protective effect of TNF-α in acute injury might be worked though inhibiting pro-inflammatory factors response and increasing anti-inflammatory factors. As hepatocellular damage was aggravated at very low degree of internal TNF-α level in CCl_4_-induced liver injury. We speculated that limited internal inflammatory factors could hardly protect hepatic cells from damage. But, how did TNF-α arouse excessive inflammatory response, and whether there were other mechanisms participating in the process of acute liver injury?

### TNF-α mediated acute liver injury may via NF-κB activation and subsequent production of anti-apoptotic genes

**Fig. 4:**
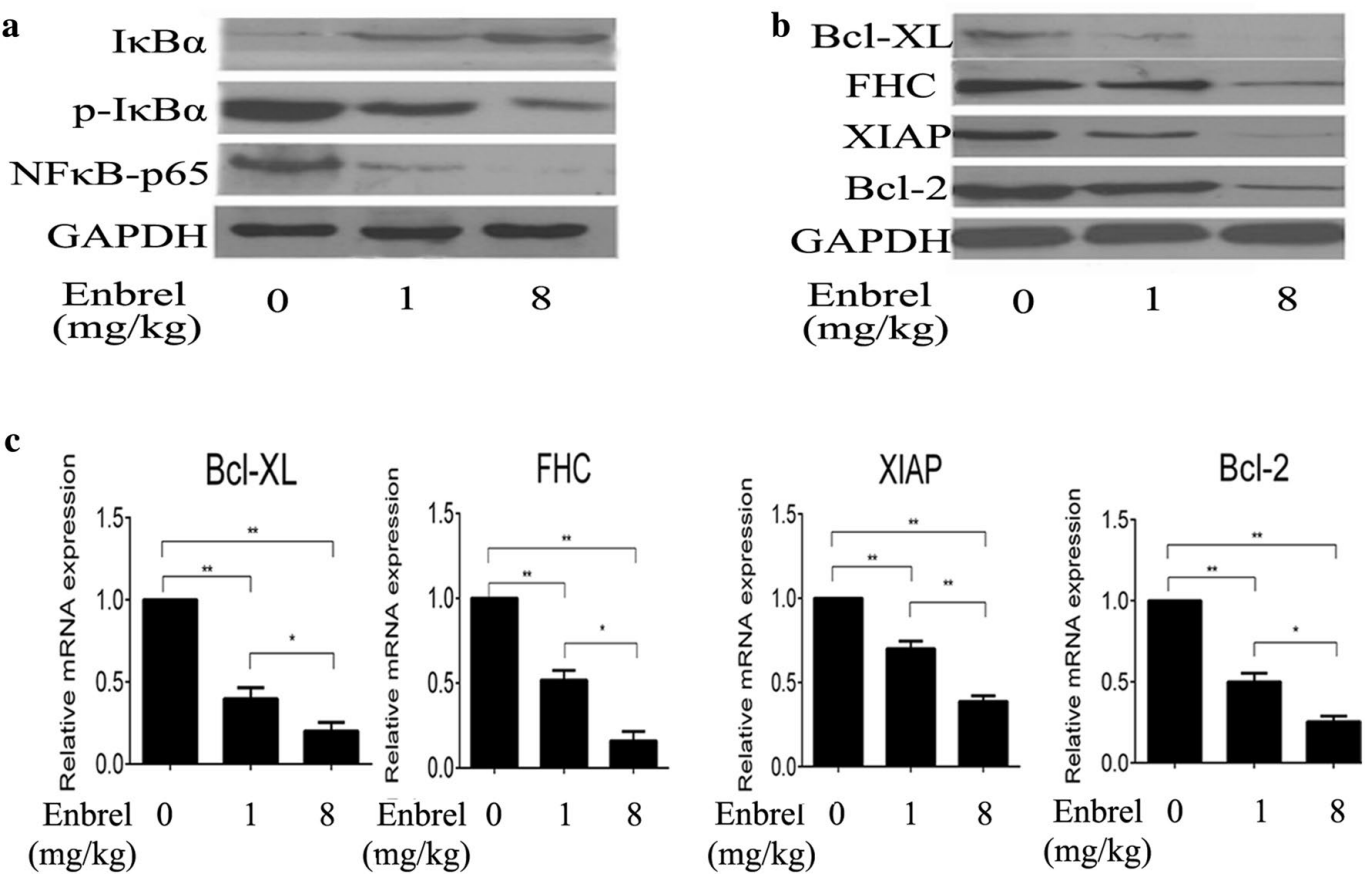
NF-κB signal pathway and anti-apoptotic gene in acute liver injured model. **a** Western-blot analyzed relevant proteins expression of NF-κB pathway include IκBα, p-IκBα and NF-κB-p65 proteins in CCl_4_-treated rats. The more doses of Enbrel were added, the lower expression of p-IκBα and NF-κB-p65 proteins they were, while the expression of IκBα had an opposite effect. **b** Western-blot and **c** RT-PCR analyzed anti-apoptosis include Bcl-XL, FHC, XIAP and Bcl-2 in CCl_4_-induced rats. As dose of Enbrel were increased, the expression of anti-apoptosis gene declined. We set mRNA expression of these genes as one in each control groups. Data are presented as mean with SD experiments. (*p < 0.05, **p < 0.01)

Previous studies have shown that TNF-α played an important role in acute liver injury. Considering that NF-κB, a crucial transcription factor, is widely accepted for regulating inflammation, innate and adaptive immunity [24, 25]. Then, we detected the relative expression of NF-κB signal pathway during the process of different levels of TNF-α in CCl_4_-induced liver injury. Western-blotting results indicated that p-IκBα (Phospho-Inhibitor of Inhibitor of KappaB) and NF-κB-p65 production were activated in CCl_4_ model. But they were inhibited as pretreatment with Enbrel, while the expression of IκBα had an opposite result (Fig. 4a). For IκBα is an inhibitor protein of NF-κB, spread outside of cell nucleus. Degradation of it by phosphorylation could activate NF-κB pathway. Meanwhile, we detected subsequent production of anti-apoptotic gene include Bcl-XL, FHC, XIAP and Bcl-2 in the CCl_4_-induced liver injury. Western-blotting and RT-PCR results showed that the expression of anti-apoptosis genes were declined as TNF-α was blocked by increased Enbrel (Fig. 4b, c). It suggested that anti-apoptosis genes were also activated in CCl_4_-induced liver acute injury and weakened as the concentration of TNF-α was down regulated. These results possibly indicated that TNF-α activated NF-κB signal pathway for further stimulating anti-apoptosis genes in protection of hepatic cells in acute liver injury.

## Discussion

Many studies have focused on the role of TNF-α as a cytokine participate the occurrence and development of liver injury [11]. Our present study provided experimental evidences that TNF-α performed protective and damaging effect at the same time in the CCl_4_-induced acute liver injury. For the protective side, our results demonstrated that the serum ALT and AST at 0 mg/kg Enbrel, a genetically engineered fusion protein which can specifically bind and inactivate TNF-α [22], is less than 8 mg/kg Enbrel (the highest level) in Fig. 1a, b. Hematoxylin and Eosin staining results also showed that the extent of hepatocellular steatosis was less at 0 than 8 mg/kg Enbrel (Fig. 1d). High level of serum TNF-α was detected at 0 mg/kg Enbrel, and very low level at 8 mg/kg Enbrel by Elisa in CCl_4_-induced liver injury (Fig. 1c). These results indicated that liver damage was slighter at 8 mg/kg Enbrel compared to the 0 mg/kg. Therefore, we believed that TNF-α had some protective effect in the CCl_4_-induced liver injury. For the damaging side, our results suggested that serum ALT and AST were decreased (Fig. 1a, b), as the Enbrel was added from 0 to 1 mg/kg in acute liver injury. And hepatocellular steatosis was also alleviated in this process. These results suggested that TNF-α also had some damaging effect in the CCl_4_-induced liver injury. Moreover, the protective effect seemed more prominent than the damaging effect at 0 mg/kg Enbrel.

On the account of apoptosis play a significant role in the injured tissues [26, 27], and is essential for clearance of potentially injurious inflammatory cells and subsequent efficient resolution of inflammation [28]. Then, we next detected hepatocellular apoptosis at different levels of TNF-α in CCl_4_-induced liver injured rats. As expected, the number of apoptotic hepatocytes were prominently decreased as the level of TNF-α was slightly down-regulated, while elevated at both excessively low and high levels of TNF-α (Fig. 2a, b). Meantime, Western-Blotting (Fig. 2c) showed the similar result: the expression of hepatocellular apoptosis was weak at 1 mg/kg Enbrel, and strong at 0 than 8 mg/kg Enbrel. In addition, the expression of apoptosis was stronger at 8 than 0 mg/kg Enbrel. These data further demonstrated that TNF-α was an indispensable cytokine and played a dual role in acute liver injury. Interestingly, in our working, we found that the change of liver function presented an obvious “V” shape in the whole process of persistently increased Enbrel. We speculated that the declined speed of damaging effect of TNF-α was faster than the protective effect during the process of persistently increasing Enbrel in the CCl_4_-induced acute liver injury. Initially, as result of injurious hepatocytes stimulated by CCl_4_, TNF-α was recruited to repair damaged organization. However, too much TNF-α initiated hepatocytes damage instead. After Enbrel was increased gradually from 0 to 1 mg/kg, TNF-α were decreased (Fig. 1c), and its damaging effect had been receded as well as the protective effect. But the damaging effect was receded more than the protective effect. Relatively, the protective effect was prominent at the maximum when Enbrel was added at 1 mg/kg, which leaded to alleviate the whole extent of liver injury. As Enbrel was continually added from 1 to 8 mg/kg, the damaging effect and the protective effect of TNF-α were further receded. Yet, the extent of protective effect was decreased more than its damaging effect. Thus, its protective effect and damaging effect were declined to the maximum and were almost abolished at 8 mg/kg Enbrel. Therefore, the injurious hepatocytes caused by CCl_4_ at very low level of TNF-α were more serious than at high level. Because the protective effect of TNF-α was almost deficient.

However, acute injury is related with inflammatory responses [29], which accompany with the change of inflammatory factors level. To further investigate the reasons for the formation of “V” shape, we detected various pro-inflammatory factors including IL-6, IL-8, IL-1β, IFN-γ as well as anti-inflammatory factors IL-4 as TNF-α was blocked in CCl_4_-induced acute liver injury. RT-PCR indicated the mRNA expressions of IL-6, IL-8, IL-1β and IFN-γ were hardly influenced in the common rats no matter how much Enbrel were pretreated (Fig. 3). However, in the acute liver injured rats, these factors were subsequently decreased when TNF-α was blocked, while IL-4 had a completely adverse change. This because IL-4, as an anti-inflammatory factor, is secreted by Th2 cell [30], and can inhibit Th1 cell producing IFN-γ, IL-6, TNF-α et al. [23]. Yet, relevant pro-inflammatory factors were decreased and meanwhile anti-inflammatory factors were correspondingly increased in the process of increasing Enbrel into CCl_4_-inducced rats. Therefore, we speculated that excessively inflammatory factors were recruited and destroyed the defensive system at 0 mg/kg Enbrel in acute CCl_4_-induced liver injured rats. Next, reduction of TNF-α could decline pro-inflammatory factors, then weakened hepatocytes damage from strong inflammatory response in acute injury. However, a certain concentration of inflammation is required for the activation of innate and adaptive immunity, which is essential for host defense [31, 32]. As TNF-α was blocked at high dose of Enbrel, pro-inflammatory factors were hardly detected. Although the damaging effect were almost abolished. But, the protective effect was also deficient, which might result in more serious hepatocellular damage. However, how did TNF-α and inflammatory factors exert the protective effect in the process of increasing Enbrel in the acute liver injury?

Some literatures have reported that the stimulation of TNF-α initiates a cascade of signaling events, which lead to the activation of NF-κB [24], whose signal pathway make an essential contribution to liver homeostasis and wound-healing processes [9, 33, 34]. Then, we further detected relative expressions of NF-κB and its subsequent productions by Western-Blotting in our study. It was excitedly that we discovered the expressions of NF-κB and subsequent production were gradually decreased as Enbrel was increased, and were hardly detected at 8 mg/kg Enbrel. Furthermore, our results showed that consistently down-regulated TNF-α concentration accompanied with declining of the expressions of antiapoptotic genes include Bcl-XL, FHC, XIAP, Bcl-2, all of which have protective effects. And their decreased extents were also dose-dependent on Enbrel. It indicated that NF-κB played a significantly role and its effect was gradually declined as well as anti-apoptotic genes, when the concentration of TNF-α was blocked by Enbrel in the CCl_4_-induced acute liver injury. Above of these findings, We speculated that the protective effect of TNF-α was emerged though NF-κB signal pathway, which further activated the antiapoptotic genes include Bcl-XL, FHC, XIAP, Bcl-2 et al., then take the protective role for the hepatocytes in the CCl_4_-induced acute liver injury. As TNF-α was blocked by Enbrel, its protective effect gradually decreased. In the initiate stage, subcutaneous injection of CCl_4_ into rats caused acute hepatocellular damage by its production of cytochrome P450 and the binding reaction with intermediate metabolites. Too much TNF-α and pro-inflammatory factors were recruited to the injured hepatocytes, then activated overexpression of NF-κB signal pathway, which further activated antiapoptotic genes to defend acute liver injury. As concentration of TNF-α was appropriately decreased from 0 to 1 mg/kg Enbrel, NF-κB was gradually weakened and the antiapoptotic genes were also activated slackly. However, the hepatocellular damage from CCl_4_ always existed as well as the damaging effect from TNF-α and inflammatory response. And the protective effect was a bit stronger than the damaging effect of TNF-α, meanwhile, its decreased speed was slower than the damaging effect. Thus, the whole of the hepatocellular damage was alleviated, and to the minimum at 1 mg/kg Enbrel. As the concentration of TNF-α was sequentially decreased from 1 to 8 mg/kg Enbrel, the protective effect from the NF-κB and antiapoptotic gene were continuously decreased. Meanwhile, the damaging effect resulted from TNF-α and inflammatory response was also weakened. In addition, the protective effect was declined faster than the damaging effect, and these effects were almost abolished at 8 mg/kg Enbrel. Thus, the hepatocellular damage was further aggravated at 8 mg/kg Enbrel, even was more serious than at 0 mg/kg Enbrel. Concurrently, the antiapoptotic genes were gradually down-regulated as TNF-α was consistently blocked by Enbrel (Fig. 4b, c), as result of the NF-κB was down-regulated. However, the hepatocellular damage induced by CCl_4_ was almost existed, and the damaging extent was the lightest at 1 mg/kg Enbrel, the most at 8 mg/kg Enbrel due to the change of the concentration of TNF-α. Thus, the number of apoptotic hepatocytes was the least at 1 mg/kg Enbrel, and the most at 8 mg/kg Enbrel (Fig. 2a, b). As the result of the protective effect of TNF-α, the hepatocellular apoptosis were slighter at 0 than at 8 mg/kg.

Our findings are clinically relevant, because moderately decrease internal TNF-α level can effectively protect liver from acute injury. But consistently down-regulated TNF-α level seems not a good choice for the treatment of acute liver injury. Meanwhile, present data shed novel mechanism on understanding different concentrations of role activating in acute liver injury caused by CCl_4_ and provide new conceptual information for therapeutic development of acute liver injury though properly reducing internal TNF-α level.

## Methods

### Animal models and treatment

All procedures involving animals were performed in accordance with the institutional animal welfare guidelines of Second Military Medical University and approved by the Ethics Committee of Eastern Hepatobiliary Surgery Hospital (Ethics Committee Approved Code:EHBHKY2012-002-7).

100 six weeks old Sprague–Dawley male rats (200–210 g) were purchased from Shanghai Experimental Animal Center of the Chinese Academy of Sciences, Shanghai, China. They were kept in ordinary cages at room temperature of 25 ± 3 °C with a 12 h dark/light cycles, and have free access to standard laboratory feed and water. To study the effects of TNF-α in acute injured rats, they were equally divided into nine groups (10 rats per group). CCl_4_ (1 ml/kg) was subcutaneously injected into Sprague–Dawley rats. Enbrel at dose of 0, 0.25, 0.5, 1, 2, 4 or 8 mg/kg was intraperitoneally administered to the CCl_4_-injected rats 15 min before the CCl_4_ injection. 24 h later, all the animals were weighted, sacrificed, collected the blood while livers were removed, weighted and perfuse in ice-cold saline solution. Liver samples were treated with liquid nitrogen and stored at −80 °C for further studies.

### Serum ALT, AST and TNF-α assay

Serum activities of ALT and aspartate aminotransferase (AST) were measured with an autoanalyzer (Spotchem Co., Kyoto, Japan). Serum levels of TNF-α in rats were measured by enzyme-linked immunosorbent assay (ELISA) using the Immunotech Human TNF-α ELISA kit (Immunotech SAS, Marseille Cedex 9, France). The results are expressed as U/L.

### Hematoxylin–eosin staining and TUNEL assay of liver sections

Liver tissues for histopathological examination were fixed with 10 % neutral buffered formalin, processed and trimmed, embedded in paraffin, sectioned to a thickness of approximately 5 μm, liver sections were stained with HE following standard protocol, then it was performed for light microscopic examination. TUNEL assay was performed using the In Situ Cell Death Detection kit (Roche Molecular Biochemicals).

### Immunohistochemistry

Quantitatively measure the expression of cleaved caspase-3 by immunohistochemical staining. Liver tissues were fixed in Carnoy, embedded in paraffin, and sectioned at 5 μm. The sections of tissues were dewaxed and rehydrated with freshly distilled water. Samples underwent inactivation of endogenous peroxidase for 10 min and were washed with distilled water for 5 min (three times), then immersed in 0.1 mol/L citrate buffer solution (pH = 6.0) and heated in a microwave oven until boiling (twice, with a 5-minute interval). After washed with phosphate buffered saline, antigen-retrieval buffers were added for 10 min and tissues were then flushed three times with PBS. At room temperature, samples were non-specifically blocked with normal goat serum for 20 min. Sections were incubated overnight at 4 °C with rabbit anti-human cleaved caspase-3 antibody. Sections were washed in PBS and incubated at room temperature for 10 min with biotinylated goat anti-rabbit IgG. After washing with PBS, sections were incubated with streptavidin-peroxidase for 10 min and then stained with diaminobenzidine for 10 min and washed for 5 min. Samples were stained with hematoxylin, and were dehydrated and mounted for microscopic examination. Images of the sections were obtained using the Image-Pro Plus 4.5 software (Media Cybernetics, Silver. Spring, USA) with brown staining under light microscopy indicating a positive reaction of cleaved caspase-3.

### Western-blot analysis

Total soluble proteins extraction from liver tissue and western-blot analysis were performed as described. Antibodies used in western-blot experiments were specific for cleaved caspase-3, IκBα (inhibitor of NF-kappa B-α), p-IκBα (Phospho-IκBα), BCL-2 (B cell leukemia/lymphoma 2), BCL-XL (B-cell lymphoma-extra large), XIAP (X-linked Inhibitor of Apoptosis Protein), FHC (Ferritin heavy chain), β-actin and goat anti-rabbit secondary antibody were obtained from Cell Signaling Technology. NFκB-p65 antibody was obtained from Abcam.

### RT-PCR analysis

The transcript levels of FHC, Bcl-XL, Bcl-2, XIAP, TNF-α, IL-1β, IFN-, IL-6, IL-8 and IL-4 in liver tissues of rats were measured using RT-PCR technique. Total RNA was isolated using the TriPure reagent (Roche Diagnostics), RNA concentrations and purity were determined by measuring the absorbance A260–A280 nm ratio. The relative quantities of mRNAs were obtained by using the comparative Ct method and were normalized with glyceraldehydes-3-phosphate dehydrogenase (GAPDH). The primer sequences were as following: FHC: Forward primer 5′-CCACGTGACCAACTTACGC-3′, Reverse primer 5′-AGTCAGCTTATCTCTCATCACCG-3′; Bcl-XL: Forward primer 5′-TCCCTTCAGAACCTTATCTTGG-3′, Reverse primer 5′-TCCCGGAAGAGTTCATTCAC-3′; Bcl-2: Forward primer 5′-ACAGCCAGGAGAAATCAAA-3′, Reverse primer 5′-GTCGCTACCGTCGTGACTTC-3′; XIAP: Forward primer 5′-CTGCATTGCATTCCATTAGC-3′, Reverse primer 5′-TGCTTCTCTGTCTAAGGTTTCAA-3′; TNF-α: Forward primer 5′-TTCTGTCTACTGAACTTGGGGGTGATCGGTCC-3′, Reverse primer 5′-GTATGAGATAGCAAATCGGCTGACGGTGTGGG-3′; IL-1β: Forward primer 5′-GGCTGCTTCCAAACCTTTGA-3′, Reverse primer 5′-GAAGACACGGATTCCATGGT-3′; IL-6: Forward primer 5′-ATGAAGTTCCTCTCTGCAAGAGAC-3′, Reverse primer 5′-CACTAGGTTTGCCGAGTAGATCTC-3′; IL-8: Forward primer 5′-AATTCTCGAGTCGCGAATGGCTGCTCAAGGCTG-3′, Reverse primer 5′-ATTACGGCCGTCGCGATTAGGCATCACTGCCTG-3′.

### Statistical analysis

All data are presented as mean ± SEM. Groups were compared by analysis of variance (ANOVA) with a posteriori contrast by least significant difference. For all analyses, p < 0.05 was considered statistically significant.

## Additional file

10.1186/s13578-016-0074-x Liver injury was aggravated in the CCl_4_-induced rats of TNF-α deficiency. (**A**) Gene sequencing diagram suggested TNF-α was successfully deleted by crispr/cas9 technique. Obviously, the five basic groups were deficient during the 205–210 gene segment of TNF-α^−/−^ rat. (**B**) Serum ALT and (**C**) AST levels were examined by Roche Diagnostic kits in Hithachi Modular P Autoanalyser. The levels of ALT and AST were significantly elevated 24 h later after 1 ml/kg CCl_4_ was subcutaneously injected into Sprague–Dawley rats 24 h later (***p < 0.001). In the TNF-α^−/−^ rats, serum of ALT and AST were elevated more than the TNF-α^+/+^ rats (*p < 0.01). (**D**) Hematoxylin-eosin–stained of liver paraffin sections analyzed that there was almost no hepatocellular steatosis in the rats without treatment of CCl_4_. And the steatosis was almost distributed all over the field of vision in the TNF-α^−/−^ rats. It was more serious than the TNF-α^+/+^ rats.
