# Supplementary figures and images for: The protective or damaging effect of Tumor necrosis factor-α in acute liver injury is concentration-dependent
Source: Cell Biosci. 2016 Feb 3;6:8. doi: 10.1186/s13578-016-0074-x (PMC4739393; doi:10.1186/s13578-016-0074-x)

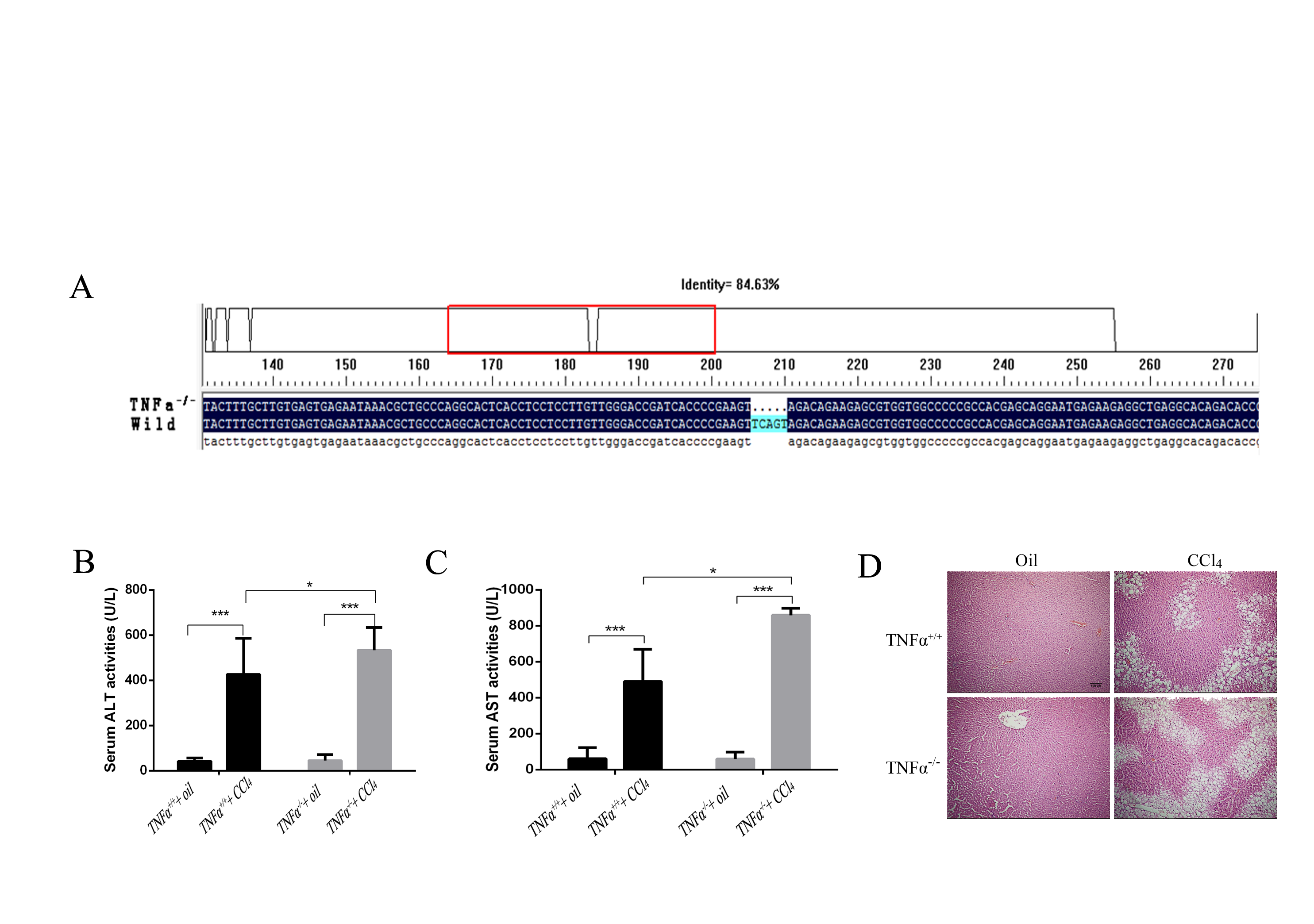

Supplement: Supplementary file 1 — 10.1186/s13578-016-0074-x Liver injury was aggravated in the CCl4-induced rats of TNF-α deficiency. (A) Gene sequencing diagram suggested TNF-α was successfully deleted by crispr/cas9 technique. Obviously, the five basic groups were deficient during the 205–210 gene segment of TNF-α−/− rat. (B) Serum ALT and (C) AST levels were examined by Roche Diagnostic kits in Hithachi Modular P Autoanalyser. The levels of ALT and AST were significantly elevated 24 h later after 1 ml/kg CCl4 was subcutaneously injected into Sprague–Dawley rats 24 h later (***p < 0.001). In the TNF-α−/− rats, serum of ALT and AST were elevated more than the TNF-α+/+ rats (*p < 0.01). (D) Hematoxylin-eosin–stained of liver paraffin sections analyzed that there was almost no hepatocellular steatosis in the rats without treatment of CCl4. And the steatosis was almost distributed all over the field of vision in the TNF-α−/− rats. It was more serious than the TNF-α+/+ rats. [file 13578_2016_74_MOESM1_ESM.tif]
